# PCR Independent Strategy-Based Biosensors for RNA Detection

**DOI:** 10.3390/bios14040200

**Published:** 2024-04-18

**Authors:** Xinran Li, Haoqian Wang, Xin Qi, Yi Ji, Fukai Li, Xiaoyun Chen, Kai Li, Liang Li

**Affiliations:** 1 Institute of Quality Standard and Testing Technology for Agro-Products, Chinese Academy of Agricultural Sciences, Beijing 100081, China; lllxinran_05@163.com (X.L.); qixin_0908@163.com (X.Q.); lifukai@caas.cn (F.L.); 2 Development Center of Science and Technology, Ministry of Agriculture and Rural Affairs, Beijing 100176, China; wanghaoqian@agri.gov.cn; 3 State Key Laboratory for Managing Biotic and Chemical Threats to the Quality and Safety of Agro-Products, Zhejiang Academy of Agricultural Sciences, Hangzhou 310021, China; jymemory12138@163.com

**Keywords:** RNA detection, PCR-free, biosensor, nanomaterial

## Abstract

RNA is an important information and functional molecule. It can respond to the regulation of life processes and is also a key molecule in gene expression and regulation. Therefore, RNA detection technology has been widely used in many fields, especially in disease diagnosis, medical research, genetic engineering and other fields. However, the current RT-qPCR for RNA detection is complex, costly and requires the support of professional technicians, resulting in it not having great potential for rapid application in the field. PCR-free techniques are the most attractive alternative. They are a low-cost, simple operation method and do not require the support of large instruments, providing a new concept for the development of new RNA detection methods. This article reviews current PCR-free methods, overviews reported RNA biosensors based on electrochemistry, SPR, microfluidics, nanomaterials and CRISPR, and discusses their challenges and future research prospects in RNA detection.

## 1. Introduction

Ribonucleic acid (RNA) is made up of phosphoric acid, ribose and base. It is usually found in biological cells as well as some viruses and viroids, and a small number of viruses are based on RNA as genetic material. RNA plays an essential role in the cells and is involved in biological processes such as protein synthesis, the regulation of gene expression and the transmission of genetic information [1,2,3]. Because RNA has many functions and meanings in biology, the detection of RNA has become particularly important. RNA detection has important applications in various fields. For example, RNA detection technology can be used in tumor diagnosis and viral infection, and the occurrence and prognosis of some diseases can be predicted by detection methods such as microRNAs (miRNAs). At the same time, RNA detection technology also plays a vital role in medical research, such as RNA sequence analysis, which can be used to study the mechanism of gene expression and transcription regulation. In addition, RNA modifications can also be detected by RNA detection techniques [4,5].

At present, the gold standard for RNA detection is still reverse transcription quantitative polymerase chain reaction (RT-qPCR). Although this method is very reliable and reasonably analytical, it requires the support of technicians and expensive instruments [6]. These problems make the nucleic acid testing technology unsuitable for its integration into miniaturized devices for clinical applications. Therefore, in order to avoid dependence on professional technicians and instruments in the process of nucleic acid testing, developing new diagnostic methods to achieve accurate, rapid and portable nucleic acid testing and quantification is urgently needed.

**Figure 1:**
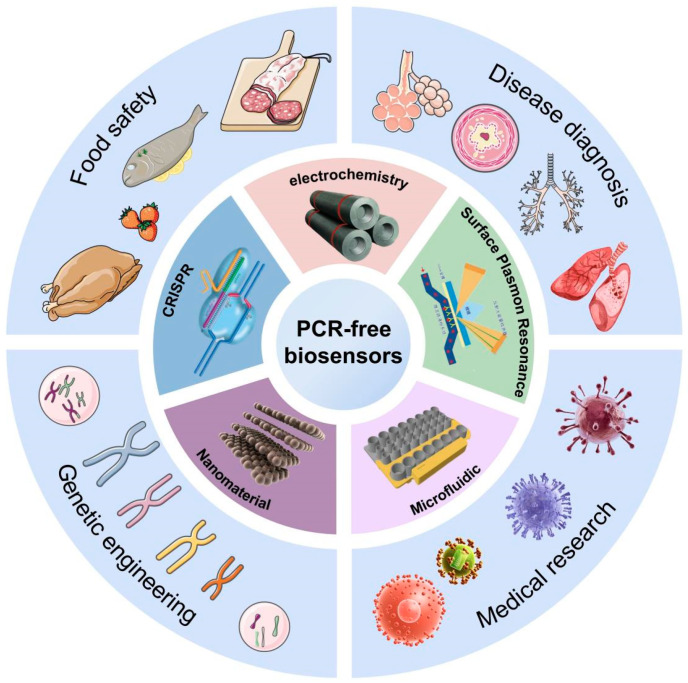
PCR-free biosensors for RNA detection. This figure provides an overview of RNA biosensors based on electrochemistry, SPR, microfluidics, nanomaterials and CRISPR, and applications in different fields such as disease diagnosis, medical research, genetic engineering, and food safety.

Among them, PCR-free methods are the most attractive solutions because they can perform molecular detection without the complexity of PCR, thereby reducing the cost of RNA detection and improving the application of nucleic acid testing [7]. Although the PCR-free-based nucleic acid testing methods address the limitations of traditional PCR methods, such as high cost, cumbersome operation and large instrument support, some limitations still need to be addressed with the transition from conventional diagnostic laboratories to portable bedside devices. So far, most of the reported reviews of RNA detection focus on direct detection without amplification [8,9,10], but there is a certain lack of sensitivity in the direct detection of RNA. Compared with direct detection without amplification, readers pay more attention to detection sensitivity. With the researchers’ continuous efforts, the limitations of traditional PCR methods have been solved in electrochemistry, surface plasma resonance (SPR), microfluidics, nanomaterials and CRISPR, and there have been successful cases; we will review these aspects (Figure 1).

## 2. Electrochemical-Based RNA Biosensors

Recently, electrochemical methods have made significant advances in the detection of clinically relevant RNA [11,12]. Most of these methods are based on the hybridization of the target RNA sequence to complementary probes (mainly DNA oligonucleotides) bound on the electrode surface. The hybridization of RNA with the probe produces a measurable electrochemical signal. Here, signal transduction depends on various factors, including the inherent electrical activity of the nucleobase, the presence of redox indicators (e.g., ferrocene, methylene blue), covalently bound redox markers (e.g., nanoparticles) or reporter enzymes (e.g., phosphatase, peroxidase) [13]. Finally, RNA is detected mainly by voltammetry, amperometry and impedance methods [14]. Electrochemical methods are promising for RNA detection due to their high sensitivity, rapid detection, cost-effectiveness and compatibility with small portability.

### 2.1. Electrochemical Biosensors for the Ultra-Sensitive Detection of RNA

One of the earlier RNA electrochemical detection methods was developed by Kosuke Mukumoto [15]. The method used ferrocenylcarbodiimide (I) to directly label messenger RNA (mRNA), which was coupled to an electrode immobilized with a DNA probe. The observed peak charge had a good correlation with the concentration of mRNA, as measured by Osteryoung Square Wave Voltammetry (SWV), which had successfully achieved electrochemical detection of labeled mRNAs, with a limit of detection (LOD) at the sub-nanogram level.

**Figure 2:**
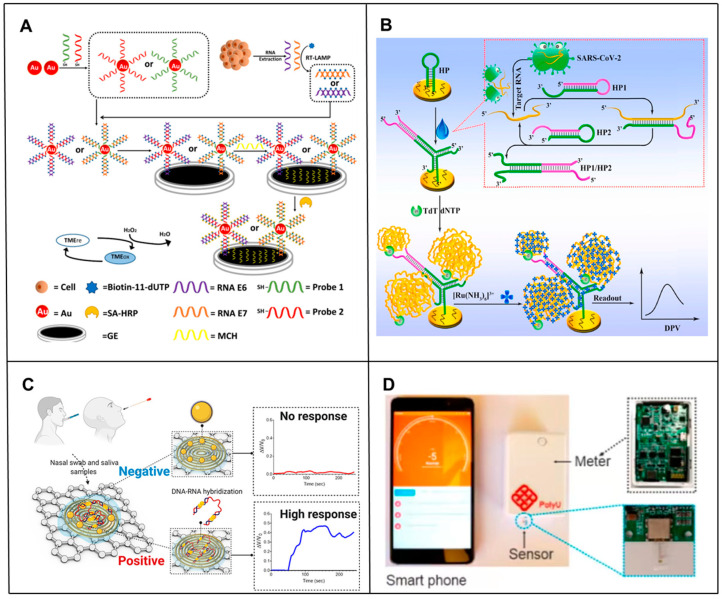
Principle of electrochemical-based RNA biosensors. (**A**) Schematic of the principle of an electrochemical biosensor based on a triple signal amplification strategy combining AuNPs, RT-LAMPs and a high-affinity biotin-affinity system for sensitive detection of mRNA [16]. (**B**) Schematic principle of an electrochemical biosensor based on CHA and TDT signal amplification for sensitive SARS-CoV-2 RNA detection [19]. (**C**) Schematic diagram of the working principle of the COVID-19 electrochemical biosensing platform [30]. (**D**) Schematic of the design of a portable biosensing platform based on organic thin film transistors. The OECT miRNA sensor is inserted into a portable meter and a smartphone is used to communicate with the portable meter via Bluetooth [34].

However, one of the biggest challenges faced by RNA biomarker detection in clinical applications is the simultaneous screening of minimal amounts of readily available RNA biomarkers in complex heterogeneous samples that may contain many non-specific targets. To address this challenge, several new approaches have been developed for high-sensitivity analysis of RNA by amplifying or using novel nanostructured electrochemical sensors. For example, Yang et al. [16] developed a triple signal amplification strategy technique combining gold nanoparticles (AuNPs), reverse transcription loop-mediated isothermal amplification (RT-LAMP), and a high-affinity biotin-affinity system to detect HPV E6/E7 mRNA. This novel signal amplification strategy exhibited a 0.1 fM (~100 copies) detection limit for HPV0 E08/E100 mRNA detection (Figure 2A). Thanyarat Chaibun et al. [17] designed a multiplex rolling circle amplification (RCA)-based electrochemical biosensor for rapid detection of nucleocapsid phosphoprotein (N gene) and Spike protein (S gene) of SARS-CoV-2 in clinical samples. Combining the high amplification capacity of RCAs with the sensitivity of electrochemical detection methods, viral N or S genes as low as 1 copy/μL could be detected within two hours, resulting in detection performance comparable to RT-qPCR in clinical samples. Zhang et al. [18] proposed a novel polymerase-assisted cyclic electrochemiluminescent aptamer biosensor for ultrasensitive leukemia marker gene mRNA detection. Combining polymerase-assisted signal amplification with AuNPs, the detection limit was 4.3 × 10^−17^ mol/L, which led to a much higher detection sensitivity. Peng et al. [19] developed an electrochemical biosensor that combined the signal amplification ability of catalytic hairpin assembly (CHA) [20,21,22,23] and terminal transferase (TDT) [23]. The electrochemical signal was significantly amplified by the electrostatic adsorption of a large number of negatively charged long single stranded DNA (ssDNA) and a large number of positively charged Ru(NH_3_)_6_^3+^, and the detection limit was as low as 26 fM. At the same time, it has been applied in complex matrices and highly stable clinical patient samples, showing great clinical application prospects (Figure 2B). Recently, an apurinic/apyrimidinic endonuclease 1 (APE1) mediated target-responsive Structure Switching Electrochemical (SSE) biosensor was developed by the Li’ group for Strawberry Mottle Virus (SMoV) RNA detection. The essence of the proposed SSE biosensor relied on the structure switching that caused the position conversion of the AP site within dsDNA and ssDNA. They used an SSE biosensor to detect target RNA, achieving a limit detection at the fM level, and successfully verified its performance in detecting SMoV in strawberry leaf-like varieties [24].

Most conventional electrochemical strategies for targeting nucleotides face tedious interfacial manipulation and washing procedures, as well as stringent reaction conditions for tool enzymes, thus limiting their potential applications. To address this problem, a series of enzyme-free electrochemical biosensors has been developed. For example, Cheng et al. [25] and Zhao et al. [26] proposed a non-enzymatic, ultrasensitive electrochemical biosensor using a hybridization chain reaction (HCR) strategy for signal amplification. For sensitive signal amplification and highly specific detection of target mRNA, ideal sensitivities with detection limits of 3 fM and 30 fM were achieved, respectively. Atie Roohizadeh et al. [27] developed an ultrasensitive label-free nano biosensor for the detection of hepatitis C virus (HCV) RNA without target denaturation. Copper oxide (CuO) and AuNPs were utilized to increase the electron transfer conductivity and reaction kinetics and improve the biosensor conductance; this strategy achieved a low detection limit of 1 fM. Emily Kerr et al. [28] studied a sensitive, rapid and portable electrochemiluminescence (ECL)-based biosensor for detecting miRNA-21. The biosensor combined turned-on ECL molecular beacons (MBs) with magnetic bead-based extraction of miRNA target sequences without the need for complex signal amplification strategies using enzymes or hairpin probes, resulting in a limit of detection up to 500 amol, which could be easily applied to point-of-care (POC) applications [29]. Overall, these methods avoid the steps of mostly enzymatic amplification of target RNA and address the problems of sample manipulation, amplification bias and longer detection time caused by the enzymatic amplification step.

### 2.2. Electrochemical Biosensors for the Rapid Detection of RNA

In addition, based on the ultra-sensitivity to RNA electrochemical detection in pursuit of rapid detection, researchers have combined nanomaterials with simple electrical readout methods. For example, Maha Alafeef et al. [30] invented a fast (less than 2 min), low-cost, quantitative paper-based electrochemical biosensor chip using AuNPs covered with highly specific antisense oligonucleotides (ssDNA) targeting the viral N gene. The device, which imparted a sensing probe to a paper-based electrochemical platform to generate nucleic acid tests, was relatively portable and fast, and its readings could be recorded with a simple handheld reader to enable digital detection of SARS-CoV-2 genetic material (Figure 2C). Ye et al. [31] designed a rapid and sensitive detection method of RNA using composite screen-printed carbon electrodes (SPCEs) modified with multi-walled carbon nanotubes (MWNTs). MWNTs displayed the catalytic properties of direct electrochemical oxidation of the adenine residues of RNA, resulting in indicator-free detection of RNA concentration. Within 5 min, the proposed method allowed for the rapid detection of yeast transfer RNA (tRNA) ranging from 8.2 μg mL^−1^ to 4.1 mg mL^−1^.

### 2.3. Electrochemical Biosensors in RNA POCT

Moreover, in order to solve the cumbersome biosensor manufacturing steps in the process of using electrochemical detection of RNA, some miniaturized and portable electrochemical biosensors have been developed successively; these generally replace traditional bulky electrodes by easy to-manufacture and miniaturized electrodes or use portable devices such as smartphones instead of traditional machines for reading. For example, Md. Nazmul Islam et al. [32] developed an amplification-free electrochemical method using screen-printed gold electrodes (SPE-Au) for the sensitive and selective detection of mRNA. Target mRNA was selectively isolated by magnetic separation and directly adsorbed onto the unmodified SPE-Au. In addition to not requiring any prior enzymatic amplification of the mRNA, it used mRNA adsorbing directly to the surface of the unmodified SPE-Au electrode, thus avoiding the cumbersome manufacturing steps of traditional biosensors. In addition, researchers have developed a simple yet fast and sensitive electrocatalytic assay for bacterial ribosomal RNA (rRNA), exploiting DNA and rRNA hybridization to the hairpin DNA probe, immobilized on the SPE-Au surface, and DNA-mediated electrocatalysis for signal amplification. The detection limit of the developed method for *E. coli* rRNA was as low as the fM level [33]. Fu et al. [34] constructed a portable and smartphone-controlled biosensing platform based on disposable organic electrochemical transistors for ultrasensitive analyses of miRNA biomarkers in less than 1 h, opening a window for low-cost mobile diagnostics of various diseases (Figure 2D). Li et al. [35] designed and prepared a portable electrochemical isothermal nucleic acid amplification test (E-INAAT) device integrating real-time monitoring and labeling-free electrochemical detection functions and a supporting plug-and-play disposable pH-sensitive potential sensor. The device, integrated with a Bluetooth module, could be implemented in smartphones for real-time monitoring of isothermal nucleic acid amplification tests (INAATs), rather than relying on heavy instruments, in the home for SARS-CoV-2 pathogens. Ultra-rapid self-inspection provides a simple, efficient and low-cost method for the development of portable, fully integrated medical detection equipment against infectious diseases.

Overall, these electrochemical biosensors are widely used in the field of nucleic acid testing due to their outstanding advantages, such as high sensitivity, simplicity of equipment, cost-effectiveness and miniaturized portability. Despite significant advances in these electrochemical methods, most of these biosensors are highly dependent on a series of optimization steps in a well-equipped laboratory setup as they are only proof-of-concept demonstrations. Several obstacles to translating these laboratory-based proof-of-concept demonstrations into real-world clinical applications exist. At this stage, our main efforts should focus on improving blocking biosensor surfaces with variously designed self-assembled monolayers or the co-immobilization of blocking molecules.

## 3. SPR-Based RNA Biosensors

Surface Plasmon Resonance (SPR), known as a label-free optical biosensor, is a direct detection method that utilizes a specific mode (surface plasmon) of a metal-dielectric waveguide to measure changes in the refractive index caused by biomolecular interactions occurring on the surface of the SPR biosensor. SPR is a highly sensitive method with many advantages, such as excellent reliability, selectivity and reproducibility. It has a wide range of applications in the real-time monitoring of biomolecular interactions and the detection of biological and chemical analytes based on labeled or unlabeled forms. Recently, the use of SPR biosensors for RNA detection studies has been reported.

### 3.1. Nanomaterial-Enhanced SPR Biosensors in RNA Detection

Based on a variety of signal amplification methods, including nanoparticle enhancement, super-sandwich assembly, streptavidin/biotin complex, antibody amplification, enzymatic reaction, triple structure formation and CHA, the limitations of SPR methods in detecting low-concentration biomolecules can be overcome, making them suitable for clinical diagnosis [36].

**Figure 3:**
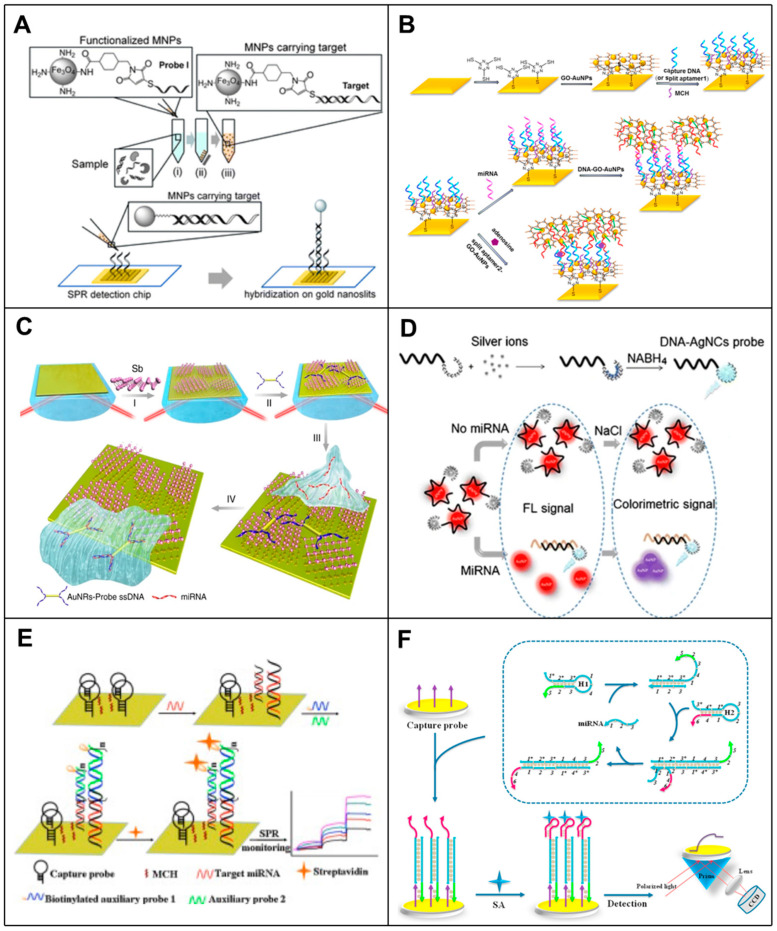
Principle of SPR-based RNA biosensors. (**A**) Schematic diagram of SPR biosensor based on functionalized MNP with gold nanoslit for mRNA detection [39]. (**B**) Schematic of SPR biosensor based on GO-AuNP composites. GO-AuNP composites were used as sensing substrate and signal amplification element [40]. (**C**) Schematic diagram of the strategy used to test the principle of antimonene-miRNA hybridization [41]. (**D**) Construction of DNA templated AgNC (DNA/AgNC) fluorescent probe for the detection of microRNA-155 and the schematic illustration of detection procedure by the FRET-based nano-biosensor [45]. (**E**) Schematic representation of miRNA detection assay using SPR biosensor based on DNA super-sandwich assemblies and streptavidin amplification [47]. (**F**) Schematic representation of miRNA SPR biosensor based on mismatched catalytic hairpin assembly amplification coupled with streptavidin aptamer [48].

Among these methods, nanotechnology has enhanced the performance and sensitivity of SPR in development. Nanoparticles can provide numerous biosensing functions and applications due to their excellent biocompatibility, large specific surface area, wide structural diversity, and significant biological simulation characteristics. As early as 2008, researchers had developed a highly sensitive detection of 16S rRNA in *E. coli* using an SPR biosensor combined with AuNPs. In this method, a cationic gold nanoparticle was synthesized by using the neutral skeleton characteristics of a peptide acid probe (PAN), and the signal was amplified by ion interaction with the 16S rRNA hybridized on the SPR biosensor chip immobilized with a PNA probe. The detection limit of this method for *E. coli* rRNA was 58.2 ± 1.37 pg mL^−1^, and *Staphylococcus aureus* could be detected without the purification of rRNA using this method [37]. Subsequently, Zhang et al. [38] constructed a highly sensitive SPR RNA biosensor using a two-dimensional metallic material called GeP_5_ nanosheets as the sensing material. Theoretical evaluations have shown that the presence of GeP_5_ nanosheets can significantly enhance the plasma electric field of Au films, thereby improving sensing sensitivity. The functionalization of GeP_5_ enabled GeP to realize nanosheets with specific complementary DNA (cDNA) probes for detecting SARS-CoV-2 RNA sequences with high sensitivity down to 10 aM and excellent selectivity. Mansoureh Z. Mousavi et al. [39] demonstrated an ultrasensitive assay for the detection of mRNA biomarkers based on SPR on functionalized magnetic nanoparticles (MNPs) intercalated with gold nanoscale. The assay used MNP to capture biomarker target molecules and then introduced the target-carrying MNP into the SPR chip to hybridize with a probe immobilized on a gold nanoslit to enhance the signal, which enabled the measurement of target molecules as low as 7 fM (equivalent to 1.26 × 10^5^ molecules) in a 30 μL sample (Figure 3A). Li et al. [40] developed a novel, sensitive and multifunctional SPR biosensor based on graphene oxide (GO)-AuNPs composites. In this biosensor, by using two layers of GO-AuNPs for signal amplification, the GO-AuNP composite was not only used as a sensing substrate but also as a signal amplification element because the AuNPs have a large specific surface area, to the extent that they can immobilize more captured DNA molecules, which amplifies the SPR response and enables the SPR biosensor to exhibit excellent sensitivity (Figure 3B). Xue et al. [41] designed an SPR biosensor based on antimony alkene two-dimensional nanomaterials to amplify the SPR signal by gold nanorods (AuNR)-conjugating ssDNA, which achieved an extremely low detection limit (amol), exceeding existing sensing methods, and quantified miRNA molecules at trace attomole levels (Figure 3C). Zhang et al. [42] presented a newly designed SPR biosensor for cytomegalovirus (CMV)-specific miRNA, utilizing the unmodified method of polyadenine [poly(A)]-Au interactions exhibiting a high affinity comparable to that of gold-sulfur (Au-S) interactions. In addition, MNPs are used for analyte separation, thus avoiding non-specific adsorption. Currently, the SPR biosensing platform has been successfully used for the multiplexed detection of CMV-related miRNA, UL22A-5p and UL112-3p, with detection limits of 112 fM for UL108A-24p and 3 fM for UL22-5p.

In addition, metal nanoparticles, such as AuNPs and AgNPs, have remarkable optical properties because the visible region has resonant surface plasma with resonant wavelengths, allowing them to display different colors depending on the wavelength, resulting in optical detection of [43] by anti-SPR biosensors. For example, G et al. [44] developed a bi-functional plasma biosensor that combined plasma photothermal (PPT) effects and local surface plasmon resonance (LSPR) sensing transduction for the clinical diagnosis of SARS-CoV-2. In this study, DNA targets were detected by nucleic acid hybridization using a two-dimensional gold nanoisland (AuNI) functionalized with cDNA receptors modified by mercapto. SARS-CoV-2’s RNA-dependent RNA polymerase (RdRp) sequence LOD was approximately 0.22 ± 0.08 pM using this LSPR-based biosensor. In a study by Yasaman-Sadat Borghei et al. [45], a dual-mode sensing system based on fluorescent DNA-modified silver nanoclusters and AuNPs was presented, which allowed naked-eye visualization of miRNA and provided rapid FRET detection. Using nanoclusters and AuNPs to transfer energy between them, the team identified and quantified miRNA in biological samples without using expensive and sophisticated instruments (Figure 3D).

### 3.2. Signal Amplification Strategy-Enhanced SPR Biosensors in RNA Detection

In addition to the high sensitivity detection of label-free optical biosensors using nanoparticles, several other methods to amplify signals have been developed, including super-sandwich assembly, streptavidin/biotin complex, antibody amplification, enzymatic reactions, triplex structure formation and catalytic hairpin assembly. For example, Wang et al. [46] developed an enzyme-free sensitive SPR biosensor based on AuNPs and DNA super-sandwich for miRNA detection using amplification of AuNPs coupled to DNA super-sandwich with a detection limit of 21 fM. Ding et al. [47] reported an SPR biosensor for nucleic acid testing. Through signal amplification-enabled sensitive nucleic acid analysis without enzyme assistance based on DNA super-sandwich assembly and the biotin/streptavidin system, this strategy was highly sensitive, and the selective detection of miRNA could detect target miRNA as low as 30 pM in 9 min and could be applied to the determination of miRNA in real samples (Figure 3E). Li et al. [48] developed an SPR biosensor coupling mismatch CHA amplification with programmable streptavidin aptamer (SA-aptamer) for the specific and highly sensitive detection of target miRNAs. Under optimal conditions, this design strategy could detect target miRNAs as low as 1 pM and was successfully applied to the determination of spiked miRNAs in human total RNA samples (Figure 3F). Li et al. [49] have developed an ultra-sensitive multiplex SPR biosensor for the quantification of a standard-free miRNA. This approach introduced a mass transfer restriction (MTL) strategy for absolute miRNA quantification. By evaluating the factors affecting the probe/target interaction (including length and structure), the MTL and quantitative detection of the miRNA were achieved with an LOD of 500 fM without any signal amplification.

It can be seen that the SPR-based detection methods only need to capture the RNA at the sensor site, and the methods are simple and highly sensitive. However, in order to achieve a high sensitivity, some signal amplification strategies must be performed. Furthermore, SPR-based sensors have made advances in reusability and miniaturization, as they usually require only one light source and one detector as a device configuration. Combined with these elements, the social implementation of SPR detection sensors will enable workers in the field to perform rapid virus detection in minutes using a combination of smartphones and simple detection kits.

## 4. Microfluidic-Based RNA Biosensors

### 4.1. Paper-Based Microfluidic RNA Biosensors

The application scenario for PCR-free RNA detection is mostly point-of-care testing (POCT) [50]. It is a rapidly growing field that involves using paper-based microfluidic devices as a tool to conduct POCTs, particularly since they come with many inherent advantages, including low cost, folding ability, ideal biocompatibility and disposability, and they are rapidly becoming more popular. Their analyzing capabilities are also attractive, including capillary force-driven sample transfer [51,52,53]. As a result of the porous structure of paper, fluid can flow through it capillarily, which is suitable for storage, mixing, flow control and the multi-analysis of reagents [54]. There is a diversity of applications and biological targets that can be studied using the type of paper, the geometry of the device and the coating of the paper used in PADs.

**Figure 4:**
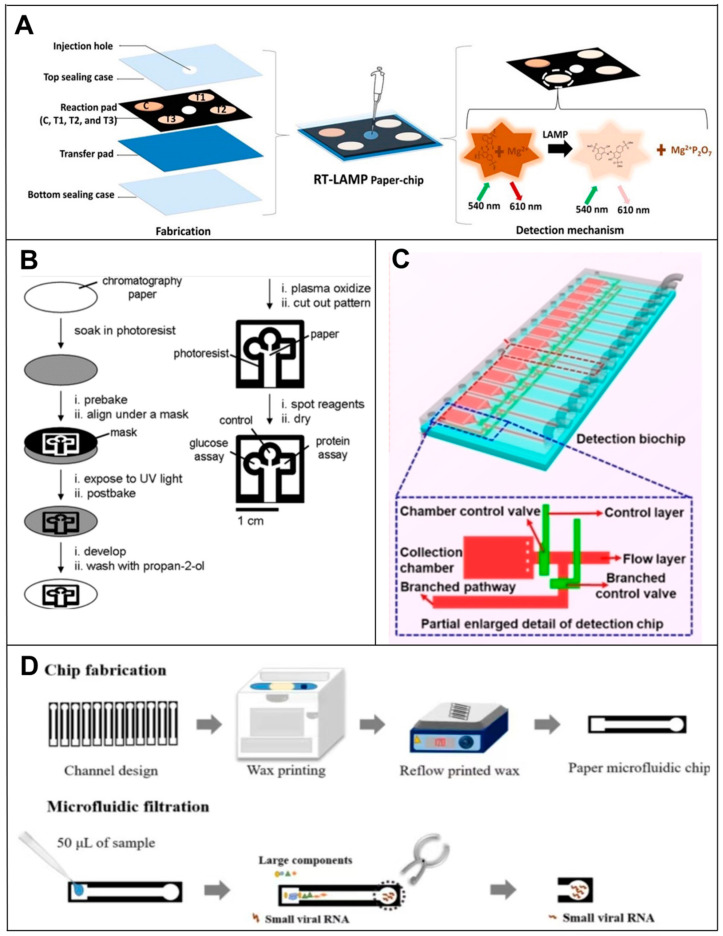
Principle of microfluidic-based RNA biosensors. (**A**) Fabrication of a paper-chip device for viral RNA amplification and detection [58]. (**B**) Principles of fabricating PADs using photolithography [52]. (**C**) Schematic representation of the microfluidic biochip structure for the detection of SARS-CoV-2 [64]. (**D**) Principle of wax printing for manufacturing PADs [62].

The lateral flow assay (LFA) is one of the main techniques used in PAD devices. Among the main components of the LFA device are a sample pad, a conjugate pad, a nitrocellulose membrane (NC) and an absorbent pad. Using strips cut into strips, these four parts are interconnected to form a one-dimensional flow [55]. After being introduced into the sample pad, the sample contains the target, which reacts with the recognition probe after passing through the conjugate pad. After passing through the NC membrane, the target-coupled probe complex passes over a control line and a test line, each containing an immobilized antibody specific to the target. Excess samples and unreacted probes are removed from the sample as it flows into the absorbent pad. Positive samples produce both the test and control lines, whereas negative samples only produce the control line. Liu’s team [56] developed a sandwich-type nucleic acid hybridization reaction using DNA probes labeled with AuNPs for the detection of miRNAs using an LFA-based paper-based microfluidic system. Upon accumulating AuNPs on the test line, a colorimetric signal was produced which could be compared with the control line visually or quantitatively using a portable strip reader. This same approach was used by Zheng et al. [57] to develop a microfluidic device for the simultaneous measurement of three miRNAs, miRNA-21, miRNA-210 and miRNA-155, using NC membranes. Bhagwan S. Batule et al. [58] demonstrated a two-step strategy for extracting and detecting viral RNA from infectious diseases within one hour. A ready-to-use device for viral RNA extraction and detection was successfully prepared using paper as a substrate. The strategy used a handheld RNA extraction paper strip device to capture and elute viral RNA (e.g., Zika, Dengue and Chikungunya), followed by an RT-LAMP assay using another paper microarray device. The entire process (extraction to detection of viral RNA) was completed in less than 1 h and was simple, sensitive and cost-effective (Figure 4A). Natalia M. Rodriguez et al. [59] developed a test strip-based assay that used a rapid, isothermal, RT-LAMP assay without the need for a thermal cycler. Sample-to-result testing could be completed in as little as 45 min at the POC and had a clinically relevant viral load LOD of 106 copies/mL, a 10-fold improvement in performance over current rapid immunoassays. The method is, therefore, suitable for rapid diagnosis, providing a simple and inexpensive platform for immediate test development.

In addition to this, there are many other fabrication techniques for μPADs. For forming hydrophobic microfluidic channels on paper substrates, many methods have been proposed for fabricating PADs. They can usually be categorized into printed and non-printed methods. Two different groups [60] demonstrated the fabrication of PADs using a solid wax printer and a hot plate back in 2009. To create a hydrophobic barrier on paper, a wax pattern is printed on the surface and melted into the paper. In a similar manner, Ashok Mulchandani et al. [61] and Kattika Kaarj et al. [62] used wax paper printers to fabricate PADs targeting the sensitive detection of miRNAs and ZIKV, respectively (Figure 4D). Even today, wax printing is the most widely used printing method for PAD fabrication due to its simplicity and low cost. It was recently demonstrated that invasive fungi can be visible and quantifiable at the point of time with a hydrogel-integrated paper-based analysis device (ReaCH-PAD) with a microfluidic scale readout and CRISPR Cas12a response. A series of enzymatic reactions is used to amplify and transduce signals using DNA hydrogels combined with a series of enzymatic reactions, as well as a paper-based microfluidic chip for visual quantitative analysis [63]. Its detection targets are 18S rRNA generic conserved fragments linked with the CRISPR Cas12a system. For non-printed fabrication methods, fluid-constrained barriers are created on the substrate by means of masters, stamps or masks instead of printing a hydrophobic barrier layout onto a paper substrate. Using this method, the Whiteside group demonstrated the simultaneous detection of glucose and proteins in 2007 [52]. A hydrophobic barrier pattern was created on the paper substrate by irradiating photoresist-impregnated paper with ultraviolet (UV) rays before baking and developing (Figure 4B). The photolithography process has high resolution and dimensional stability, but it is susceptible to lateral spreading after the hot plate heating step, resulting in a loss of resolution.

### 4.2. Microchip-Based RNA Biosensors

As mentioned above, μPADs enable rapid, low-cost and sensitive nucleic acid testing analysis, which is promising for POC disease diagnosis and on-site molecular testing. However, because one of the challenges of paper-based devices is usually analytical sensitivity, and because they also bring disadvantages, such as cross-reactions, false positive signals and even environmental pollution, researchers have developed other more accurate and environmentally friendly device-chip microfluidic devices.

For example, Han’s team [64] invented a microfluidic biochip for the rapid and ultrasensitive detection of SARS-CoV-2 by taking advantage of the specific SARS-CoV-2 RNA and probe DNA reactions in the microfluidic channel and fluorescence signaling modulation by nanomaterials, which enabled the ultrasensitive optical detection of SARS-CoV-2 RNA without the need for a molecular amplification step (Figure 4C). Qin et al. [65] proposed an NoV digital isothermal detection (NoV-DID) chip based on a gas-driven microfluidic chamber which uses a simple monolayer of polydimethylsiloxane (PDMS) for the detection of NoV GII.4. Combined with reverse transcription recombinase-assisted amplification (RT-RAA), it overcomes the limitations of the NoV detection technology and effectively reduces time, cost and dependence on instruments. In contrast to methods using reverse transcription, Zhang et al. [66] reported a new microfluidic RNA microarray (MIRC, a prototype of microchips) strategy based on the genomic replication of DNA polymerase-extended RNA primers on DNA templates with dNTP [67,68], which allowed the direct detection of RNAs without the need for reverse transcription, thus overcoming the tedious reverse transcription process. The method is characterized by rapid detection (within 20 min), high sensitivity, automation and high throughput [69,70]. In addition, the introduction of a microfluidic chip reduces reaction time, reagent usage and assay complexity.

Therefore, compared with RNA detection by PCR, highly miniaturized microfluidic technologies can integrate complex nucleic acid detection processes on a piece of paper or a chip [71,72], thus reducing the complexity of the operation and helping to build an automated and efficient diagnostic system [73,74,75,76]. Especially in the last two years, with the huge demand for POCT for COVID-19 testing, highly miniaturized microfluidic devices have provided essential tools for integrating complex nucleic acid testing processes and will increasingly become the trend and backbone of pandemic disease response.

## 5. Nanomaterial-Based RNA Biosensors

Since viral RNA detection and identification involves longer operating times and greater device complexity, there is a great need to identify alternative viral detection targets and procedures for a simpler and more rapid diagnosis. Generally speaking, nanomaterials are usually used in vitro in combination with other methods to amplify signals and improve sensitivity. However, due to their own properties, such as the quenching of graphene oxide and the fluorescence of carbon nanotubes themselves, nanomaterials can be used to detect RNA [77,78], but individual detection is usually achieved in the cell. Based on this, nanomaterials as a separate analytical tool also provide a feasible alternative to RT-PCR for rapid and accurate virus detection.

### 5.1. Graphene Oxide-Based RNA Biosensors

**Figure 5:**
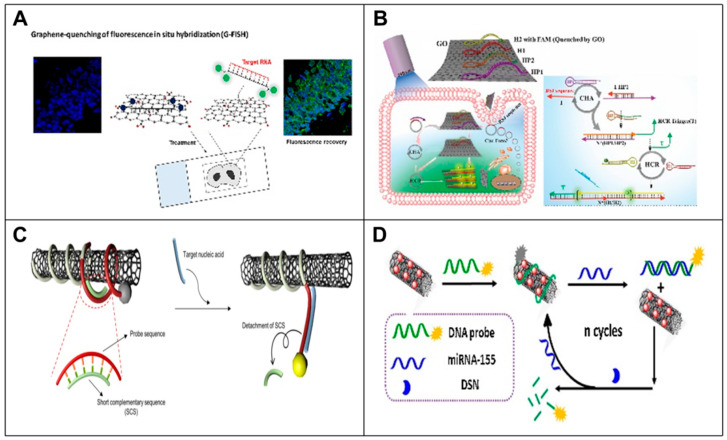
Principle of nanomaterial-based RNA biosensors. (**A**) GO nanoplatform for non-coding RNA detection in FFPE tissue specimens. Schematic of G-FISH [80]. (**B**) Schematic representation of a GO-based CHA and HCR signal dual amplification system for circ-Foxo3 imaging in living cells [81]. (**C**) A fluorescence-based CNT-DNA biosensor using a SCS. The schematic diagram of the improved CNT-DNA biosensor with an SCS improves the sensitivity of detecting the target miRNA [85]. (**D**) Schematic diagram of MWCNT/AuNCs used as a novel fluorescence bursting platform for miRNA-155 detection [86].

Graphene oxide (GO) is a single-atom-thick two-dimensional carbon nanomaterial with properties such as large specific surface area, biocompatibility and effective fluorescence burst. Taking advantage of these promising properties, some methods for the direct detection of RNA based on GO have been developed. For example, Jiang et al. [79] reported a multiplexed GO fluorescent nanoprobe for the intracellular detection and quantification of mRNA in living cells by utilizing the fluorescence bursting property of GO. The detection limit of this GO-based nanoprobe was as low as 0.26 nM for mRNA mimics, and the nanoprobe was able to simultaneously perform relative quantification and intracellular detection of multiple target mRNAs in living cells compared to conventional mRNA detection methods. Do Won Hwang et al. [80] developed a robust nanoprobe platform for the simultaneous quantification and intracellular detection of multiple target mRNAs in living cells using GO quenching and fluorescence in situ hybridization (G-FISH). They also explored in situ hybridization recovery for sensitive RNA detection in formalin-fixed paraffin-embedded (FFPE) tissues (Figure 5A). Li et al. [81] presented a novel GO-based CHA and HCR signal dual amplification system (GO-CHA-HCR, or GO-AR) for circ-Foxo3 imaging detection in living cells. This method enabled the detection limit of circ-Foxo3 to be as low as 15 pM with excellent sensitivity and selectivity (Figure 5B). Yang et al. [82] developed a highly sensitive strategy for live cell and in vivo miRNA fluorescence imaging detection based on MB with GO enhanced signaling molecular bursts. The detection limit was as low as 30 pM in the presence of miRNA. This simple and effective strategy provided a new sensing platform for highly sensitive detection and simultaneous imaging analysis of multiple low-level biomarkers in living cells and in vivo.

### 5.2. Carbon Nanotube-Based RNA Biosensors

Carbon nanotubes (CNTs) are a widely used nanomaterial. In addition to properties such as a large specific surface area and carrying an abundance of electrons, the high accessibility of CNTs and easy-to-use fluorescence analyses allow them to be used as a material for the direct detection of RNA [83,84]. For example, Shrute Kannappan et al. [85] reported a fluorine-based CNT-DNA biosensor by introducing short complementary sequences (SCSs) that could regulate the binding strength of the probe sequence to CNT, thereby enhancing its reactivity to target oligonucleotides. The introduction of SCSs significantly increased the LOD of the biosensor, and this strategy could also be used to multiplex a set of miRNAs for a range of other pathological states by redesigning the probe sequence and measuring the corresponding fluorescence in a very short time (~1 h) (Figure 5C). Ma et al. [86] developed a sensitive sensing platform for the detection of a potential marker of breast cancer miRNA-155 [87,88] based on multiwall carbon nanotube-gold nanocomposites (MWCNT/AuNCs) as a new platform of fluorescence quenching coupled with DSN-assisted recovery signal amplification (Figure 5D).

Nanotechnology is likely to play an important role in the continued development of PCR-free methods for RNA detection. PCR-free methods are especially valuable in developing countries and resource-constrained settings. In order to progress in this field, cutting-edge innovations in nanotechnology will be essential, including nanoparticles, GO and DNA nanostructures, as well as nanomaterials such as CNTs, nanowires and quantum dots. This innovation and development will benefit PCR-free nucleic acid testing methods.

## 6. CRISPR-Based RNA Biosensors

CRISPR (clusters of regularly interspaced short palindromic repeats) was first discovered in the 1980s and is thought to be an adaptive immune system coupled to Cas proteins which uses RNA-directed nucleases to cleave invading nucleic acids [89], thereby allowing resistance to invading exogenous DNA and viruses in bacteria and archaea. The system is divided into two categories [90], with the most studied Cas9, Cas12 and Cas13 all belonging to the second category. In addition to being used for gene editing, Cas12 and Cas13 are involved in non-specific ssDNA or RNA cleavage after specific recognition of the target (trans-cleavage activity), making CRISPR-Cas a promising tool for the detection of nucleic acids [91,92]. Abnormal expression and mutation of RNA may be harmful to cells and cause disease, so the detection of abnormally expressed RNA or disease-related RNA mutations provides an avenue for disease diagnosis [93,94]. However, the detection of most disease-associated RNAs is demanding in terms of accuracy and detection limit due to low abundance and high sequence similarity among family members. The low tolerance of the CRISPR-Cas system to base mismatches in target nucleic acid sequences gives it excellent recognition of single-base mismatches. Therefore, CRISPR-Cas-based biosensors have a broad application prospect in RNA detection.

### 6.1. CRISPR-Cas9-Based RNA Biosensors

**Figure 6:**
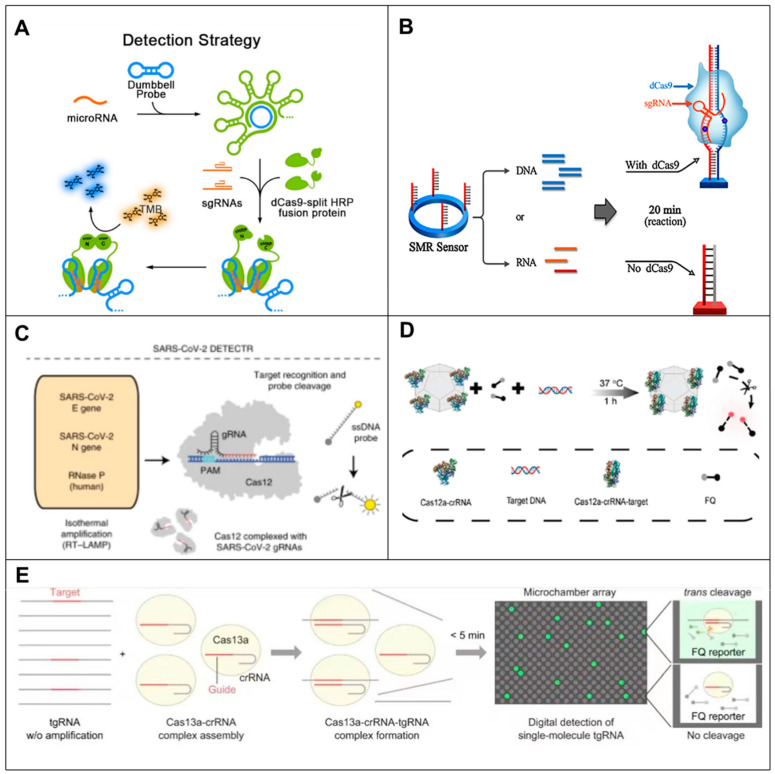
Principle of CRISPR-based RNA biosensors. (**A**) Schematic representation of the RCA-CRISPR-split-HRP (RCH) method based on RCA, CRISPR-Cas9 and cleaved-root peroxidase technologies for miRNA detection [95]. (**B**) Schematic of CRISPR/dCas9-mediated biosensor. SMR biosensor, silicon mirroring resonator biosensor [99]. (**C**) Schematic of SARS-CoV-2 DETECTR workflow [106]. (**D**) Cas12a assembled with MAF-7 to form nanobiocomposites with higher stability and trans-cleavage activity for nucleic acid testing [111]. (**E**) Schematic illustration of SATORI. LwaCas13a–crRNA–tgRNA cleaves FQ reporters, leading to fluorescence increases in a microchamber array device [116].

In recent years, some researchers have been trying to expand the application of CRISPR-Cas9 to the field of RNA detection. Cas9 has been used as a tool to detect miRNAs by converting RNA targets into substrates capable of triggering CRISPR-Cas9 responses. For example, Qiu et al. [95] were the first to perform miRNA detection using CRISPR-Cas9. The assay system incorporated isothermal amplification, detection and reporting based on RCA, CRISPR-Cas9 and split-horseradish peroxidase technologies. First, the miRNA was sequentially converted into a large DNA fragment containing multiple repeating complementary sequences and random neck loop structures of the dCas9 target by RCA amplification reaction. The Split-HRP-dCas9 protein recognized and localized to the RCA product under the guidance of specific small guide RNAs (sgRNAs), which subsequently led to the formation of active horseradish peroxidase (HRP), catalyzing the oxidation of tetramethylbenzidine (TMB). This method enabled the detection of trace miRNA in samples with single base specificity (Figure 6A). Wang et al. [96] developed an miRNA biosensor consisting of dCas9, miRNA-mediated sgRNA and red fluorescent protein. The biosensor provided an example for measuring miRNA activity and tracking cell state transitions in order to allow timely monitoring of miRNA activity in stem cell differentiation and cancer progression. Moe Hirosawa et al. [97] designed an miRNA-responsive AcrllA4 switch based on the expression of endogenous miRNA activity-controlled *S. pyogenes* Cas4 inhibitor AcrllA9, which, together with Cas9 or dCas9-VPR guide RNA complex, indirectly activates Cas9, enabling multiple intracellular miRNA sensing. By sensing intracellular miRNAs, this system could provide a powerful tool for future therapeutic applications and genome engineering.

In addition to detecting miRNA, there are some reports of the CRISPR-CAS system in the detection of mRNA and viral RNA. For example, Li et al. [98] designed an mRNA CRISPR biosensor that activated the cleavage function of Cas9 by switching the blocked sgRNA with the target RNA. In this strategy, mRNA-sensing CRISPR was constructed by guide RNA (gRNA) reconstitution and toe-mediated strand shift, in which each target site could be controlled independently. Experiments have shown that the switch could be embedded into the gRNA and used as an RNA biosensor, which could orthogonally detect multiple mRNA inputs and provide CRISPR/Cas9 response outputs. Bonhan Koo et al. [99] developed an improved molecular diagnostic tool that utilized a CRISPR/dCas9-mediated biosensor to couple dCas9 and a single micro-ring resonator biosensor for label-free and real-time detection of pathogenic RNA, achieving single-molecule sensitivity for RNA detection and 100-fold more sensitivity than RT-PCR detection. It improved the sensitivity and specificity of pathogen diagnosis in clinical samples (Figure 6B). Tin Marsic et al. [100] devised a method utilizing CRISPR/Cas9 enzymes for DNA scanning and recognition and VirD2 release covalently binding to ssDNA probes for LFA conjugation for SARS-CoV-2 viral RNA detection. The method employed a chimeric fusion between dCas9 and VirD2 in combination with an ssDNA reporter as a detection complex. A sensitive, specific and low-cost detection method was realized. In addition, CRISPR/cas9-based tools have been used as antiviral drugs for the treatment of HIV infections and for the detection of Zika virus and methicillin-resistant Staphylococcus aureus infections.

### 6.2. CRISPR-Cas12-Based RNA Biosensors

The Cas12 protein is a member of the CRISPR family and can be programmed with CRISPR-deriver RNA (crRNA) to specifically bind to complementary ssDNA and double stranded DNA (dsDNA) targets [101,102]. Cas12 is an alternative to Cas9 due to its unique properties, such as the ability to target T-rich motifs and the absence of the need for trans-activation crRNA. Along with specific double strand breaks (DSBs), Cas12a also undergoes non-specific cleavage on other ssDNA molecules. These non-specific tendencies are triggered only when the crRNA binds to its complementary target, known as trans-cleavage activity [103]. However, Cas12a has weak trans-cleavage activity, making nucleic acid testing less sensitive [91,104]. When combined with preamplification, cas12a-mediated detection can detect concentrations as low as 2 aM [104,105]. For example, James P. Broughton et al. [106] developed a CRISPR-cas12-based lateral flow assay for the detection of viral infection [107] called SARS-CoV-2 DNA Endonuclease Targeting CRISPR Trans Reporter (DETECTR). This method relied on the trans-cleavage activity of Cas12a proteins activated after Cas12a recognition of the target RNA. In addition, the LAMP step was combined with the DETECTR technology of CRISPR-Cas12 to enrich the target sequences. The purpose of rapid (30–40 min) detection, easy implementation and high accuracy of SARS-CoV-2 in clinical samples was achieved, providing a visual and faster alternative for RT-PCR detection (Figure 6C). Shi-Yuan Li et al., using the characteristic [108] of non-targeting ssDNA during the formation of Cas12a/crRNA/target DNA ternary complex, developed a low-cost multi-purpose efficient detection system HOLMES (one-hour low-cost multi-purpose highly efficient system), which could be used for the rapid and low-cost detection of target RNA [109]. At the same time, Cas12a-based HOLMES can also detect nucleic acids with aM sensitivity. Compared to Cas12a, Cas12b exhibited higher activity against the exes. Subsequently, Liang et al. developed an updated version of HOLMESV2 that combined Cas2b and isothermal amplification to detect nucleotides, distinguish single nucleotide polymorphisms (SNPs), and quantify dsRNA and RNA methylation [110]. Unlike the DETECTR, which is only used for qualitative measurements, HOLMES can be used for quantitative detection. A successful attempt was made to create an active Cas12a nanocomposite that could be used as a biosensor without shell deconstruction or enzyme release.

Instead of using preamplification to achieve signal amplification, Zhi Run Ji et al. [111] proposed a strategy to use metal-organic frameworks (MOFs) to protect Cas12a from harsh environments and successfully constructed an active Cas12a nanocomposite, Cas12a-on-MAF-7 (COM), as a biosensor for the first time, without the need of isothermal amplification. This strategy achieved an ultra-sensitive SARS-CoV-2 RNA detection with a detection limit of one copy and solved the problem of poor stability of CRISPR-Cas12a (Figure 6D).

### 6.3. CRISPR-Cas13-Based RNA Biosensors

CRISPR-Cas13a is the only CRISPR effector that targets RNA and has the ability to go beyond signal amplification [112]. One of the milestones in using CRISPR-Cas for RNA detection was the discovery by Zhang’s team in 2016 of the trans-cleavage activity of Cas13a (also known as C2c2) [92]. The target RNA is specifically recognized by crRNA and subsequently cleaved by Cas13a [113]. When used together, activated Cas13a utilizes its unique trans-cleavage activity, the RNA provided by the lateral cleavage RNA probe, and the fluorophore quencher labeled RNA reporter with trans-cleavage activity [103], which could be used together to determine specific target sequences. The CRISPR-Cas13 system has great potential for detecting viral RNA due to its reliability, high sensitivity and ease of implementation [114]. RNA can be directly detected by the side branch cleavage of CRISPR-Cas13a. For example, Alexandra East-Seletsky et al. [115] described a method for the direct detection of RNA using Cas13a trans-cleavage. By designing fluorophore quencher labeled RNA reporters, known as reporter genes, they achieved efficient signal amplification detection of target RNAs with pM sensitivity, as each activated Cas13a is capable of cleaving thousands of reporter genes. Subsequently, Hajime Shinoda et al. developed a platform that enables the accurate and rapid detection of single stranded RNA (ssRNA) at the single-molecule level, the CRISPR-based Amplification-Free Digital RNA Assay (SATORI) platform. The combination of CRISPR-Cas13-based RNA detection technology and microchamber array technology avoids the long detection time and false negative or false positive results due to amplification errors caused by the preamplification process in CRISPR-based methods, resulting in a maximum sensitivity of 10 fM for the detection of ssRNA targets with high specificity and a short detection time (less than 5 min) [116] (Figure 6E).

Despite the high specificity and simplicity of direct detection of RNA with Cas13a, the abundance of RNA in organisms is particularly low. To further improve sensitivity, incorporating nucleic acid amplification is an effective strategy. Max J. Kellner et al. recently established a CRISPR-based diagnostic platform that combines nucleic acid preamplification with the CRISPR-Cas13 system for specific recognition of the desired RNA sequence. The platform, known as Specific High Sensitivity Enzyme Reporter Unlock (SHERLOCK), allows for multiplexed, portable and ultra-sensitive RNA detection from clinically relevant samples. However, a drawback of SHERLOCK made it unsuitable for RNA quantitative detection [105]. In the following year, Gootenberg et al. upgraded SHERLOCK version 2 (SHERLOCKv2) [105] to allow simultaneous detection of multiple targets. The SHERLOCKv2 is a powerful tool for nucleic acid testing because of its increased sensitivity and quantitative and visual readouts. CRISPR-Cas13a cascade-based viral RNA detection in clinical samples was reported by Yuxi Wang et al. as a label-free, isothermal method [117]. SARS-CoV-2 RNA was directly detected using Cas13a/crRNA to activate transcriptional amplification for light-up RNA aptamer output [118,119,120]. This assay achieved high sensitivity for SARS-CoV-2 RNA detection at a detection limit of 0.216 fM by integrating Cas13a/crRNA’s RNA-specific recognition capability and trans-cleavage activity into cascade amplification.

As a whole, the CRISPR-Cas system has the potential to be a valuable tool for nucleic acid testing [121,122,123,124]. A molecular diagnosis of SARS-CoV-2 is mainly performed using RT-qPCR, which is time-consuming and expensive. Further, patients may experience anxiety, irritability and fear as a result of false positives produced by RT-qPCR. A CRISPR-based approach, on the other hand, eliminates expensive probes (e.g., quenchers and fluorescent-modified RNA probes) and expensive equipment (e.g., thermal circulators), thus reducing detection complexity and equipment costs [125,126,127,128]. Furthermore, the CRISPR-Cas system can detect pathogens more rapidly in food due to its highly accurate and efficient characteristics. This significantly improves the efficiency of the food safety detection process. Additionally, CRISPR technology can identify various pollutants in food and monitor food safety conditions [129,130].

## 7. Discussion

RNA detection has important applications in various fields. In particular, the COVID-19 pandemic has introduced the need for new accurate and efficient diagnostic tools for SARS-CoV-2 RNA detection. Therefore, it is essential to choose the proper detection method. Most detection techniques are still based on PCR and RT-PCR. While PCR-based techniques are highly sensitive and specific, their analysis requires a variety of equipment and technicians and can only be performed in laboratories. To overcome this limitation, considerable efforts have been made to perform RNA detection, and various improved or innovative PCR-free methods have been developed, such as electrochemical methods, SPR, microfluidic devices, nanotechnology and CRISPR-based detection techniques, to name a few. They overcome the limitations of PCR-based detection of RNA, and since they do not require expensive reagents and instruments, the application of PCR-free detection methods may help reduce the cost of RNA detection and thus improve the applicability of RNA detection. However, there are also some problems in the existing technologies (Table 1). For example, PCR-free-based biosensors are faced with technical problems such as miniaturization, portability, high precision and low energy consumption, which limit their popularization and development. At the same time, the PCR-free-based biosensors also have limitations in their reusability and signal interference. In addition, PCR-free-based biosensors involve multidisciplinary cross-integration, such as materials science, computers, communications, bioinformatics, biochips, etc., and require more innovation and cooperation to achieve technological breakthroughs. In conclusion, with the rapid development of new technologies and methods, we believe that more excellent and efficient detection methods will be developed in the future, which will provide scientists and clinicians with more choices. At the same time, the most economical and optimal choice can only be obtained by weighing the advantages and disadvantages of various detection methods according to the specific purpose.

## Figures and Tables

**Table 1:** Summary of PCR-free-based biosensors.

System	Combination	Sensitivity	Time	Target	Ref.
Electrochemical-based RNA biosensors	AuNPs/RT-LAMP/high affinity biotin-avidin system	0.1 fmol·L^−1^	~1 h	mRNA	[6]
	RCA	1 copy/μL	<2 h	viral N or S genes	[7]
	AuNPs/polymerase-assisted signal amplification	4.3 × 10^−17^ mol/L	<1 h	mRNA	[8]
	CHA/TDT	26 fmol·L^−1^	<1 h	SARS-CoV-2 RNA	[9]
	HCR	3 fmol·L^−1^	<1 h	mRNA	[14]
	CuO/AuNPs	1 fmol·L^−1^	~1 h	HCV RNA	[16]
	MWNTs/SPE	8.2 μg mL^−1^	<5 min	tRNA	[19]
	SPE-Au	fmol·L^−1^	<1 h	mRNA	[20]
SPR-based RNA biosensors	GeP5	10 amol·L^−1^	<1 h	SARS-CoV-2 RNA	[24]
	MNPs/AuNPs	7 fmol·L^−1^	~1 h	mRNA	[25]
	Antimonene two-dimensional nanomaterials/AuNR	amol·L^−1^	~1 h	miRNA	[27]
	MNP	3 fmol·L^−1^	~2 h	miRNA	[28]
	DNA-AgNCs/AuNPs	fmol·L^−1^	<2 h	miRNA	[31]
	AuNPs/DNA super-sandwich	21 fmol·L^−1^	~1 h	miRNA	[32]
	DNA super-sandwich/biotin-streptavidin system	30 pmol·L^−1^	<9 min	miRNA	[33]
	CHA/streptavidin aptamer	1 pmol·L^−1^	<1 h	miRNA	[34]
	MTL	500 fmol·L^−1^	~1 h	miRNA	[35]
Microfluidic-based RNA biosensors	AuNPs	fmol·L^−1^	~2 h	miRNA	[40]
	RT-LAMP	fmol·L^−1^	<1 h	Viral RNA	[42]
	RT-LAMP	160 copies/μL	<45 min	Viral RNA	[43]
	NoV-DID/PDMS/RT-RAA	fmol·L^−1^	~1 h	Viral RNA	[48]
Nanomaterial-based RNA biosensors	GO	0.26 nmol·L^−1^	<2 h	mRNA	[61]
	GO/CHA/HCR	15 pmol·L^−1^	<2 h	circRNA	[63]
	GO/MB	30 pmol·L^−1^	~1 h	miRNA	[64]
	MWCNT/AuNCs	33.4 fmol·L^−1^	~1 h	miRNA	[68]
CRISPR-based RNA biosensors	RCA/CRISPR-Cas9	fmol·L^−1^	<1 h	miRNA	[77]
	DETECTR	fmol·L^−1^	30–40 min	SARS-CoV-2 RNA	[88]
	HOLMES	amol·L^−1^	~1 h	Viral RNA	[90]
	CRISPR-Cas12/MoFs	1 copy	~1 h	SARS-CoV-2 RNA	[93]
	SATORI	10 fmol·L^−1^	<5 min	SARS-CoV-2 RNA	[97]
	SHERLOCK	fmol·L^−1^	~1 h	SARS-CoV-2 RNA	[86]
	SHERLOCKv2	fmol·L^−1^	~1 h	SARS-CoV-2 RNA	[86]
	CRISPR-Cas13	0.216 fmol·L^−1^	~1 h	SARS-CoV-2 RNA	[99]

## Data Availability

The data presented in this study are available on request from the corresponding authors.

## Abbreviations

AgNPs	Silver nanoparticles
AuNIs	gold nanoisland
AuNPs	gold nanoparticles
AuNR	gold nanorods
Au-S	gold-sulfur
cDNA	complementary DNA
CHA	catalytic hairpin assembly
CMV	cytomegalovirus
CNTs	carbon nanotubes
COM	Cas12a-on-MAF-7
CRISPR	clusters of regularly interspaced short palindromic repeats
crRNA	CRISPR-deriver RNA
CuO	copper oxide
DETECTR	DNA Endonuclease Targeting CRISPR Trans Reporter
DNA-AgNC	DNA-silver nanocluster
DSBs	double strand breaks
dsDNA	double stranded DNA
E-INAATs	electrochemical isothermal nucleic acid amplification tests
ELC	electrochemiluminescence
FFPE	formalin-fixed paraffin-embedded
G-FISH	graphene oxide-fluorescence in situ hybridization
GO	graphene oxide
gRNA	Guide RNA
HCR	hybridization chain reaction
HCV	hepatitis C virus
HOLMES	one-hour low-cost multipurpose highly efficient system
HRP	horseradish peroxidase
INAATs	isothermal nucleic acid amplification tests
LFA	lateral flow assay
LOD	limit of detection
LSPR	local surface plasmon resonance
MB	molecular beacons
miRNAs	microRNAs
MNPs	magnetic nanoparticles
MOFs	metal-organic frameworks
mRNA	messenger RNA
MTL	mass transfer restriction
MWCNT/AuNCs	multiwall carbon nanotube-gold nanocomposites
MWNTs	multi-walled carbon nanotubes
NC	nitrocellulose
N gene	nucleocapsid phosphoprotein
PAD	Paper-based microfludics
PDMS	polydimethylsiloxane
POC	point-of-care
POCT	point-of-care testing
Poly(A)	polyadenine
PPT	plasma photothermal
PAN	Peptide acid probe
rRNA	ribosomal RNA
RCA	rolling circle amplification
RdRp	RNA-dependent RNA polymerase
RT-LAMP	reverse transcription loop-mediated isothermal amplification
RT-qPCR	reverse transcription quantitative polymerase chain reaction
RT-RAA	reverse-transcription recombinase-assisted amplification
SA-aptamer	streptavidin aptamer
SCS	short complementary sequences
S gene	Spike protein
sgRNA	Small guide RNA
SHERLOCK	Specific High Sensitivity Enzyme Reporter Unlock
SNPs	single nucleotide polymorphisms
SPCEs	screen-printed carbon electrodes
SPE-Au	screen-printed gold electrodes
SPR	surface plasma resonance
ssRNA	single stranded RNA
SWV	Square Wave Voltammetry
TDT	terminal transferase
TMB	tetramethylbenzidine
tRNA	transfer RNA
UV	ultraviolet rays
μPADs	Microfluidic paper-based analytical devices

## References

[1] Mortimer S.A., Kidwell M.A., Doudna J.A. (2014). Insights into RNA Structure and Function from Genome-Wide Studies. Nat. Rev. Genet..

[2] Cui L., Ma R., Cai J., Guo C., Chen Z., Yao L., Wang Y., Fan R., Wang X., Shi Y. (2022). RNA Modifications: Importance in Immune Cell Biology and Related Diseases. Signal Transduct. Target. Ther..

[3] Laurent G.S., Shtokalo D., Tackett M.R., Yang Z., Vyatkin Y., Milos P.M., Seilheimer B., McCaffrey T.A., Kapranov P. (2013). On the Importance of Small Changes in RNA Expression. Methods.

[4] Kellner S., Burhenne J., Helm M. (2010). Detection of RNA Modifications. RNA Biol..

[5] Motorin Y., Lyko F., Helm M. (2010). 5-Methylcytosine in RNA: Detection, Enzymatic Formation and Biological Functions. Nucleic Acids Res..

[6] Ma X., Xu J., Zhou F., Ye J., Yang D., Wang H., Wang P., Li M. (2022). Recent Advances in PCR-Free Nucleic Acid Detection for SARS-CoV-2. Front. Bioeng. Biotechnol..

[7] Calorenni P., Leonardi A.A., Sciuto E.L., Rizzo M.G., Lo Faro M.J., Fazio B., Irrera A., Conoci S. (2023). PCR-Free Innovative Strategies for SARS-CoV-2 Detection. Adv. Healthc. Mater..

[8] Shinoda H., Lida T., Makino A., Yoshimura M., Lshikawa J., Ando J., Murai K., Sugiyama K., Muramoto Y., Nakano M. (2022). Automated Amplification-Free Digital RNA Detection Platform for Rapid and Sensitive SARS-CoV-2 Diagnosis. Commun. Biol..

[9] Ueda T., Shinoda H., Makino A., Yoshimure M., Lida T., Watanabe R. (2023). Purification/Amplification-Free RNA Detection Platform for Rapid and Multiplex Diagnosis of Plant Viral Infections. Anal. Chem..

[10] Wang D., Wang X., Ye F., Zou J., Qu J., Jiang X. (2023). An Integrated Amolification-Free Digital Crispr/Cas-Assisted Assay for Single Molecule Detection of RNA. ACS Nano.

[11] Tavallaie R., Darwish N., Hibbert D.B., Gooding J.J. (2015). Nucleic-Acid Recognition Interfaces: How the Greater Ability of RNA Duplexes to Bend towards the Surface Influences Electrochemical Sensor Performance. Chem. Commun..

[12] Labib M., Sargent E.H., Kelley S.O. (2016). Electrochemical Methods for the Analysis of Clinically Relevant Biomolecules. Chem. Rev..

[13] Hartman M.R., Ruiz R.C.H., Hamada S., Xu C., Yancey K.G., Yu Y., Han W., Luo D. (2013). Point-of-Care Nucleic Acid Detection Using Nanotechnology. Nanoscale.

[14] Johnson B.N., Mutharasan R. (2014). Biosensor-Based Microrna Detection: Techniques, Design, Performance, and Challenges. Analyst.

[15] Mukumoto K., Nojima T., Sato S., Waki M., Takenaka S. (2007). Direct Modification of mRNA by Ferrocenyl Carbodiimide and Its Application to Electrochemical Detection of mRNA. Anal. Sci..

[16] Yang N., Liu P., Cai C., Zhang R., Sang K., Shen P., Huang Y., Lu Y. (2021). Triple Signal Amplification Strategy for the Ultrasensitive Electrochemical Detection of Human Papillomavirus 16 E6/E7 mRNA. Enzym. Microb. Technol..

[17] Chaibun T., Puenpa J., Ngamdee T., Boonapatcharoen N., Athamanolap P., O’Mullane A.P., Vongpunsawad S., Poovorawan Y., Lee S.Y., Lertanantawong B. (2021). Rapid Electrochemical Detection of Coronavirus SARS-CoV-2. Nat. Commun..

[18] Zhang M., Zhou F., Zhou D., Chen D., Hai H., Li J. (2019). An Aptamer Biosensor for Leukemia Marker mRNA Detection Based on Polymerase-Assisted Signal Amplification and Aggregation of Illuminator. Anal. Bioanal. Chem..

[19] Peng Y., Pan Y., Sun Z., Li J., Yi Y., Yang J., Li G. (2021). An Electrochemical Biosensor for Sensitive Analysis of the SARS-CoV-2 RNA. Biosens. Bioelectron..

[20] Dai W., Zhang J., Meng X., He J., Zhang K., Cao Y., Wang D., Dong H., Zhang X. (2018). Catalytic Hairpin Assembly Gel Assay for Multiple and Sensitive Microrna Detection. Theranostics.

[21] Li P., Wei M., Zhang F., Su J., Wei W., Zhang Y., Liu S. (2018). Novel Fluorescence Switch for Microrna Imaging in Living Cells Based on Dnazyme Amplification Strategy. ACS Appl. Mater. Interfaces.

[22] Li P., Wei M., Zhang F., Su J., Wei W., Zhang Y., Liu S. (2017). Application of Spectral Crosstalk Correction for Improving Multiplexed Microrna Detection Using a Single Excitation Wavelength. Anal. Chem..

[23] Xu E., Feng Y., Yang H., Li P., Kong L., Wei W., Liu S. (2021). Ultrasensitive and Specific Multi-Mirna Detection Based on Dual Signal Amplification. Sens. Actuators B Chem..

[24] Li K., Chen T., Wang M., Li F., Qi X., Song X., Fan L., Li L. (2024). Ape1 Mediated Target-Responsive Structure Switching Electrochemical (SSE) Biosensor for RNA Detection. Sens. Actuators B Chem..

[25] Cheng Y.-H., Liu S.-J., Jiang J.-H. (2021). Enzyme-Free Electrochemical Biosensor Based on Amplification of Proximity-Dependent Surface Hybridization Chain Reaction for Ultrasensitive mRNA Detection. Talanta.

[26] Zhao T., Zhang H.-S., Tang H., Jiang J.-H. (2017). Nanopore Biosensor for Sensitive and Label-Free Nucleic Acid Detection Based on Hybridization Chain Reaction Amplification. Talanta.

[27] Roohizadeh A., Ghaffarinejad A., Salahandish R., Omidinia E. (2020). Label-Free Rna-Based Electrochemical Nanobiosensor for Detection of Hepatitis C. Curr. Res. Biotechnol..

[28] Kerr E., Farr R., Doeven E.H., Nai Y.H., Alexander R., Guijt R.M., Prieto-Simon B., Francis P.S., Dearnley M., Hayne D.J. (2021). Amplification-Free Electrochemiluminescence Molecular Beacon-Based Microrna Sensing Using a Mobile Phone for Detection. Sens. Actuators B Chem..

[29] Bhaiyya M., Pattnaik P.K., Goel S.D. (2021). A Brief Review on Miniaturized Electrochemiluminescence Devices: From Fabrication to Applications. Curr. Opin. Electrochem..

[30] Alafeef M., Dighe K., Moitra P., Pan D. (2020). Rapid, Ultrasensitive, and Quantitative Detection of SARS-CoV-2 Using Antisense Oligonucleotides Directed Electrochemical Biosensor Chip. ACS Nano.

[31] Ye Y., Ju H. (2005). Rapid Detection of Ssdna and RNA Using Multi-Walled Carbon Nanotubes Modified Screen-Printed Carbon Electrode. Biosens. Bioelectron..

[32] Islam N., Gopalan V., Haque H., Masud M.K., Al Hossain S., Yamauchi Y., Nguyen N.-T., Lam A.K.-Y., Shiddiky M.J. (2017). A Pcr-Free Electrochemical Method for Messenger RNA Detection in Cancer Tissue Samples. Biosens. Bioelectron..

[33] Jamal R.B., Vitasovic T., Gosewinkel U., Ferapontova E.E. (2023). Detection of E. Coli 23s Rrna by Electrocatalytic “Off-on” DNA Beacon Assay with Femtomolar Sensitivity. Biosens. Bioelectron..

[34] Fu Y., Wang N., Yang A., Xu Z., Zhang W., Liu H., Law H.K.-W., Yan F. (2021). Ultrasensitive Detection of Ribonucleic Acid Biomarkers Using Portable Sensing Platforms Based on Organic Electrochemical Transistors. Anal. Chem..

[35] Li Q., Li Y., Gao Q., Jiang C., Tian Q., Ma C., Shi C. (2022). Real-Time Monitoring of Isothermal Nucleic Acid Amplification on a Smartphone by Using a Portable Electrochemical Device for Home-Testing of SARS-CoV-2. Anal. Chim. Acta.

[36] Jebelli A., Oroojalian F., Fathi F., Mokhtarzadeh A., de la Guardia M. (2020). Recent Advances in Surface Plasmon Resonance Biosensors for Micrornas Detection. Biosens. Bioelectron..

[37] Joung H.-A., Lee N.-R., Lee S.K., Ahn J., Shin Y.B., Choi H.-S., Lee C.-S., Kim S., Kim M.-G. (2008). High Sensitivity Detection o 16s Rrna Using Peptide Nucleic Acid Probes and a Surface Plasmon Resonance Biosensor. Anal. Chim. Acta.

[38] Chang S., Liu L., Mu C., Wen F., Xiang J., Zhai K., Wang B., Wu L., Nie A., Shu Y. (2023). An Ultrasensitive Spr Biosensor for RNA Detection Based on Robust Gep5 Nanosheets. J. Colloid Interface Sci..

[39] Mousavi M.Z., Chen H.-Y., Wu S.-H., Peng S.-W., Lee K.-L., Wei P.-K., Cheng J.-Y. (2013). Magnetic Nanoparticle-Enhanced Spr on Gold Nanoslits for Ultra-Sensitive, Label-Free Detection of Nucleic Acid Biomarkers. Analyst.

[40] Li Q., Wang Q., Yang X., Wang K., Zhang H., Nie W. (2017). High Sensitivity Surface Plasmon Resonance Biosensor for Detection of Microrna and Small Molecule Based on Graphene Oxide-Gold Nanoparticles Composites. Talanta.

[41] Xue T., Liang W., Li Y., Sun Y., Xiang Y., Zhang Y., Dai Z., Duo Y., Wu L., Qi K. (2019). Ultrasensitive Detection of Mirna with an Antimonene-Based Surface Plasmon Resonance Sensor. Nat. Commun..

[42] Chang Y.-F., Chou Y.-T., Cheng C.-Y., Hsu J.-F., Su L.-C., Ho J.-A.A. (2021). Amplification-Free Detection of Cytomegalovirus Mirna Using a Modification-Free Surface Plasmon Resonance Biosensor. Anal. Chem..

[43] Camarca A., Varriale A., Capo A., Pennacchio A., Calabrese A., Giannattasio C., Almuzara C.M., D’Auria S., Staiano M. (2021). Emergent Biosensing Technologies Based on Fluorescence Spectroscopy and Surface Plasmon Resonance. Sensors.

[44] Qiu G., Gai Z., Tao Y., Schmitt J., Kullak-Ublick G.A., Wang J. (2020). Dual-Functional Plasmonic Photothermal Biosensors for Highly Accurate Severe Acute Respiratory Syndrome Coronavirus 2 Detection. ACS Nano.

[45] Borghei Y.-S., Hosseini M., Ganjali M.R., Ju H. (2018). Colorimetric and Energy Transfer Based Fluorometric Turn-on Method for Determination of Microrna Using Silver Nanoclusters and Gold Nanoparticles. Microchim. Acta.

[46] Wang Q., Liu R., Yang X., Wang K., Zhu J., He L., Li Q. (2016). Surface Plasmon Resonance Biosensor for Enzyme-Free Amplified Microrna Detection Based on Gold Nanoparticles and DNA Supersandwich. Sens. Actuators B Chem..

[47] Ding X., Yan Y., Li S., Zhang Y., Cheng W., Cheng Q., Ding S. (2015). Surface Plasmon Resonance Biosensor for Highly Sensitive Detection of Microrna Based on DNA Super-Sandwich Assemblies and Streptavidin Signal Amplification. Anal. Chim. Acta.

[48] Li J., Lei P., Ding S., Zhang Y., Yang J., Cheng Q., Yan Y. (2016). An Enzyme-Free Surface Plasmon Resonance Biosensor for Real-Time Detecting Microrna Based on Allosteric Effect of Mismatched Catalytic Hairpin Assembly. Biosens. Bioelectron..

[49] Li K., An N., Wu L., Wang M., Li F., Li L. (2024). Absolute Quantification of Micrornas Based on Mass Transport Limitation under a Laminar Flow Spr System. Biosens. Bioelectron..

[50] Kulkarni M.B., Goel S. (2023). Mini-Thermal Platform Integrated with Microfluidic Device with on-Site Detection for Real-Time DNA Amplification. Biotechniques.

[51] Martinez A.W., Phillips S.T., Whitesides G.M. (2010). Devices (UPADS)-Are a New Platform Designed for Assured. Anal. Chem..

[52] Martinez A.W., Phillips S.T., Butte M.J., Whitesides G.M. (2007). Patterned Paper as a Platform for Inexpensive, Low-Volume, Portable Bioassays. Angew. Chem..

[53] Kumari M., Gupta V., Kumar N., Arun R.K. (2024). Microfluidics-Based Nanobiosensors for Healthcare Monitoring. Mol. Biotechnol..

[54] Yamada K., Shibata H., Suzuki K., Citterio D. (2017). Toward Practical Application of Paper-Based Microfluidics for Medical Diagnostics: State-of-the-Art and Challenges. Lab Chip.

[55] Noviana E., Ozer T., Carrell C.S., Link J.S., McMahon C., Jang I., Henry C.S. (2021). Microfluidic Paper-Based Analytical Devices: From Design to Applications. Chem. Rev..

[56] Gao X., Xu H., Baloda M., Gurung A.S., Xu L.-P., Wang T., Zhang X., Liu G. (2014). Visual Detection of Microrna with Lateral Flow Nucleic Acid Biosensor. Biosens. Bioelectron..

[57] Zheng W., Yao L., Teng J., Yan C., Qin P., Liu G., Chen W. (2018). Lateral Flow Test for Visual Detection of Multiple Micrornas. Sens. Actuators B Chem..

[58] Batule B.S., Seok Y., Kim M.-G. (2020). Based Nucleic Acid Testing System for Simple and Early Diagnosis of Mosquito-Borne RNA Viruses from Human Serum. Biosens. Bioelectron..

[59] Rodriguez N.M., Linnes J.C., Fan A., Ellenson C.K., Pollock N.R., Klapperich C.M. (2015). Based RNA Extraction, in Situ Isothermal Amplification, and Lateral Flow Detection for Low-Cost, Rapid Diagnosis of Influenza a (H1N1) from Clinical Specimens. Anal. Chem..

[60] Carrilho E., Martinez A.W., Whitesides G.M. (2009). Understanding Wax Printing: A Simple Micropatterning Process for Paper-Based Microfluidics. Anal. Chem..

[61] Shen Y., Mulchandani A. (2023). Affordable Paper-Based Swnts Field-Effect Transistor Biosensors for Nucleic Acid Amplification-Free and Label-Free Detection of Micro RNAs. Biosens. Bioelectron. X.

[62] Kaarj K., Akarapipad P., Yoon J.-Y. (2018). Simpler, Faster, and Sensitive Zika Virus Assay Using Smartphone Detection of Loop-Mediated Isothermal Amplification on Paper Microfluidic Chips. Sci. Rep..

[63] Huang D., Ni D., Fang M., Shi Z., Xu Z. (2021). Microfluidic Ruler-Readout and Crispr Cas12a-Responded Hydrogel-Integrated Paper-Based Analytical Devices (Mreach-Pad) for Visible Quantitative Point-of-Care Testing of Invasive Fungi. Anal. Chem..

[64] Chu Y., Qiu J., Wang Y., Wang M., Zhang Y., Han L. (2022). Rapid and High-Throughput SARS-CoV-2 RNA Detection without RNA Extraction and Amplification by Using a Microfluidic Biochip. Chem.—A Eur. J..

[65] Qin Z., Xiang X., Xue L., Cai W., Gao J., Yang J., Liang Y., Wang L., Chen M., Pang R. (2021). Development of a Novel Raa-Based Microfluidic Chip for Absolute Quantitative Detection of Human Norovirus. Microchem. J..

[66] Zhang S., Chen J., Liu D., Hu B., Luo G., Huang Z. (2022). A Novel Microfluidic RNA Chip for Direct, Single-Nucleotide Specific, Rapid and Partially-Degraded RNA Detection. Talanta.

[67] Burgers P.M.J., Kunkel T.A. (2017). Eukaryotic DNA Replication Fork. Annu. Rev. Biochem..

[68] McHenry C.S. (2011). DNA Replicases from a Bacterial Perspective. Annu. Rev. Biochem..

[69] Tian T., Bi Y., Xu X., Zhu Z., Yang C. (2018). Integrated Paper-Based Microfluidic Devices for Point-of-Care Testing. Anal. Methods.

[70] Basiri A., Heidari A., Nadi M.F., Fallahy M.T.P., Nezamabadi S.S., Sedighi M., Saghazadeh A., Rezaei N. (2021). Microfluidic Devices for Detection of RNA Viruses. Rev. Med. Virol..

[71] Gao D., Ma Z., Jiang Y. (2022). Recent Advances in Microfluidic Devices for Foodborne Pathogens Detection. TrAC Trends Anal. Chem..

[72] Chen Y., Liu Y., Shi Y., Ping J., Wu J., Chen H. (2020). Magnetic Particles for Integrated Nucleic Acid Purification, Amplification and Detection without Pipetting. TrAC Trends Anal. Chem..

[73] Chen Y., Zong N., Ye F., Mei Y., Qu J., Jiang X. (2022). Dual-Crispr/Cas12a-Assisted Rt-Raa for Ultrasensitive SARS-CoV-2 Detection on Automated Centrifugal Microfluidics. Anal. Chem..

[74] Chen Y., Mei Y., Jiang X. (2021). Universal and High-Fidelity DNA Single Nucleotide Polymorphism Detection Based on a Crispr/Cas12a Biochip. Chem. Sci..

[75] Zong N., Gao Y., Chen Y., Luo X., Jiang X. (2022). Automated Centrifugal Microfluidic Chip Integrating Pretreatment and Molecular Diagnosis for Hepatitis B Virus Genotyping from Whole Blood. Anal. Chem..

[76] Chen Y., Mei Y., Zhao X., Jiang X. (2020). Reagents-Loaded, Automated Assay That Integrates Recombinase-Aided Amplification and Cas12a Nucleic Acid Detection for a Point-of-Care Test. Anal. Chem..

[77] Pinals R.L., Ledesma F., Yang D., Navarro N., Jeong S., Pak J.E., Kuo L., Chuang Y.-C., Cheng Y.-W., Sun H.-Y. (2021). Rapid SARS-CoV-2 Spike Protein Detection by Carbon Nanotube-Based near-Infrared Nanosensors. Nano Lett..

[78] Kumar S., Singh H., Feder-Kubis J., Nguyen D.D. (2023). Recent Advances in Nanobiosensors for Sustainable Healthcare Applications: A Systematic Literature Review. Environ. Res..

[79] Jiang H., Li F.-R., Li W., Lu X., Ling K. (2018). Multiplexed Determination of Intracellular Messenger RNA by Using a Graphene Oxide Nanoprobe Modified with Target-Recognizing Fluorescent Oligonucleotides. Microchim. Acta.

[80] Hwang D.W., Choi Y.R., Kim H., Park H.Y., Kim K.W., Kim M.Y., Park C.-K., Lee D. (2019). Graphene Oxide-Quenching-Based Fluorescence in Situ Hybridization (G-Fish) to Detect RNA in Tissue: Simple and Fast Tissue RNA Diagnostics. Nanomed. Nanotechnol. Biol. Med..

[81] Li H., Zhang B., He X., Zhu L., Zhu L., Yang M., Huang K., Luo H., Xu W. (2021). Intracellular Circrna Imaging and Signal Amplification Strategy Based on the Graphene Oxide-DNA System. Anal. Chim. Acta.

[82] Yang L., Liu B., Wang M., Li J., Pan W., Gao X., Li N., Tang B. (2018). A Highly Sensitive Strategy for Fluorescence Imaging of Microrna in Living Cells and in Vivo Based on Graphene Oxide-Enhanced Signal Molecules Quenching of Molecular Beacon. ACS Appl. Mater. Interfaces.

[83] Harvey J.D., Jena P.V., Baker H.A., Zerze G.H., Williams R.M., Galassi T.V., Roxbury D., Mittal J., Heller D.A. (2017). A Carbon Nanotube Reporter of Microrna Hybridization Events in Vivo. Nat. Biomed. Eng..

[84] Kim K., Yoon S., Chang J., Lee S., Cho H.H., Jeong S.H., Jo K., Lee J.H. (2020). Multifunctional Heterogeneous Carbon Nanotube Nanocomposites Assembled by DNA-Binding Peptide Anchors. Small.

[85] Kannappan S., Chang J., Sundharbaabu P.R., Heo J.H., Sung W.-K., Ro J.C., Kim K.K., Rayappan J.B.B., Lee J.H. (2022). DNA-Wrapped Cnt Sensor for Small Nucleic Acid Detection: Influence of Short Complementary Sequence. BioChip J..

[86] Ma H., Xue N., Li Z., Xing K., Miao X. (2018). Ultrasensitive Detection of Mirna-155 Using Multi-Walled Carbon Nanotube-Gold Nanocomposites as a Novel Fluorescence Quenching Platform. Sens. Actuators B Chem..

[87] Zhu W., Qin W., Atasoy U., Sauter E.R. (2009). Circulating Micrornas in Breast Cancer and Healthy Subjects. BMC Res. Notes.

[88] Mattiske S., Suetani R.J., Neilsen P.M., Callen D.F. (2012). The Oncogenic Role of Mir-155 in Breast Cancer. Cancer Epidemiol. Biomark. Prev..

[89] Wu H., Chen X., Zhang M., Wang X., Chen Y., Qian C., Wu J., Xu J. (2021). Versatile Detection with Crispr/Cas System from Applications to Challenges. TrAC Trends Anal. Chem..

[90] Safari F., Hatam G., Behbahani A.B., Rezaei V., Barekati-Mowahed M., Petramfar P., Khademi F. (2020). Crispr System: A High-Throughput Toolbox for Research and Treatment of Parkinson’s Disease. Cell. Mol. Neurobiol..

[91] Chen J.S., Ma E., Harrington L.B., Da Costa M., Tian X., Palefsky J.M., Doudna J.A. (2018). Crispr-Cas12a Target Binding Unleashes Indiscriminate Single-Stranded Dnase Activity. Science.

[92] Abudayyeh O.O., Gootenberg J.S., Konermann S., Joung J., Slaymaker I.M., Cox D.B.T., Shmakov S., Makarova K.S., Semenova E., Minakhin L. (2016). C2c2 Is a Single-Component Programmable Rna-Guided Rna-Targeting Crispr Effector. Science.

[93] Wang S., Wei S., Wang S., Zhu X., Lei C., Huang Y., Nie Z., Yao S. (2018). Chimeric DNA-Functionalized Titanium Carbide Mxenes for Simultaneous Mapping of Dual Cancer Biomarkers in Living Cells. Anal. Chem..

[94] Wang S., Song W., Wei S., Zeng S., Yang S., Lei C., Huang Y., Nie Z., Yao S. (2019). Functional Titanium Carbide Mxenes-Loaded Entropy-Driven RNA Explorer for Long Noncoding RNA Pca3 Imaging in Live Cells. Anal. Chem..

[95] Qiu X.-Y., Zhu L.-Y., Zhu C.-S., Ma J.-X., Hou T., Wu X.-M., Xie S.-S., Min L., Tan D.-A., Zhang D.-Y. (2018). Highly Effective and Low-Cost Microrna Detection with Crispr-Cas9. ACS Synth. Biol..

[96] Wang X.-W., Hu L.-F., Hao J., Liao L.-Q., Chiu Y.-T., Shi M., Wang Y. (2019). A Microrna-Inducible Crispr–Cas9 Platform Serves as a Microrna Sensor and Cell-Type-Specific Genome Regulation Tool. Nat. Cell Biol..

[97] Hirosawa M., Fujita Y., Saito H. (2019). Cell-Type-Specific Crispr Activation with Microrna-Responsive Acrlla4 Switch. ACS Synth. Biol..

[98] Li Y., Teng X., Zhang K., Deng R., Li J. (2019). RNA Strand Displacement Responsive Crispr/Cas9 System for mRNA Sensing. Anal. Chem..

[99] Koo B., Kim D.-E., Kweon J., Jin C.E., Kim S.-H., Kim Y., Shin Y. (2018). Crispr/Dcas9-Mediated Biosensor for Detection of Tick-Borne Diseases. Sens. Actuators B Chem..

[100] Marsic T., Ali Z., Tehseen M., Mahas A., Hamdan S., Mahfouz M. (2021). Vigilant: An Engineered Vird2-Cas9 Complex for Lateral Flow Assay-Based Detection of SARS-CoV2. Nano Lett..

[101] Aman R., Mahas A., Mahfouz M. (2020). Nucleic Acid Detection Using Crispr/Cas Biosensing Technologies. ACS Synth. Biol..

[102] Pickar-Oliver A., Gersbach C.A. (2019). The Next Generation of Crispr–Cas Technologies and Applications. Nat. Rev. Mol. Cell Biol..

[103] Li Y., Li S., Wang J., Liu G. (2019). Crispr/Cas Systems Towards Next-Generation Biosensing. Trends Biotechnol..

[104] Gootenberg J.S., Abudayyeh O.O., Kellner M.J., Joung J., Collins J.J., Zhang F. (2018). Multiplexed and Portable Nucleic Acid Detection Platform with Cas13, Cas12a, and Csm6. Science.

[105] Gootenberg J.S., Abudayyeh O.O., Lee J.W., Essletzbichler P., Dy A.J., Joung J., Verdine V., Donghia N., Daringer N.M., Freije C.A. (2017). Nucleic Acid Detection with Crispr-Cas13a/C2c2. Science.

[106] Broughton J.P., Deng X., Yu G., Fasching C.L., Servellita V., Singh J., Miao X., Streithorst J.A., Granados A., Sotomayor-Gonzalez A. (2020). Crispr-Cas12-Based Detection of SARS-CoV-2. Nat. Biotechnol..

[107] Mustafa M.I., Makhawi A.M. (2021). Sherlock and Detectr: Crispr-Cas Systems as Potential Rapid Diagnostic Tools for Emerging Infectious Diseases. J. Clin. Microbiol..

[108] Li S.-Y., Cheng Q.-X., Liu J.-K., Nie X.-Q., Zhao G.-P., Wang J. (2018). Crispr-Cas12a Has Both Cis-and Trans-Cleavage Activities on Single-Stranded DNA. Cell Res..

[109] Li S.-Y., Cheng Q.-X., Wang J.-M., Li X.-Y., Zhang Z.-L., Gao S., Cao R.-B., Zhao G.-P., Wang J. (2018). Crispr-Cas12a-Assisted Nucleic Acid Detection. Cell Discov..

[110] Liang M., Li Z., Wang W., Liu J., Liu L., Zhu G., Karthik L., Wang M., Wang K.-F., Wang Z. (2019). A Crispr-Cas12a-Derived Biosensing Platform for the Highly Sensitive Detection of Diverse Small Molecules. Nat. Commun..

[111] Ji Z., Zhou B., Shang Z., Liu S., Li X., Zhang X., Li B. (2023). Active Crispr-Cas12a on Hydrophilic Metal–Organic Frameworks: A Nanobiocomposite with High Stability and Activity for Nucleic Acid Detection. Anal. Chem..

[112] Tian T., Shu B., Jiang Y., Ye M., Liu L., Guo Z., Han Z., Wang Z., Zhou X. (2020). An Ultralocalized Cas13a Assay Enables Universal and Nucleic Acid Amplification-Free Single-Molecule RNA Diagnostics. ACS Nano.

[113] Gao G., Zhu X., Lu B. (2021). Development and Application of Sensitive, Specific, and Rapid Crispr-Cas13-Based Diagnosis. J. Med. Virol..

[114] Shan Y., Zhou X., Huang R., Xing D. (2019). High-Fidelity and Rapid Quantification of Mirna Combining Crrna Programmability and Crispr/Cas13a Trans-Cleavage Activity. Anal. Chem..

[115] East-Seletsky A., O’Connell M.R., Knight S.C., Burstein D., Cate J.H.D., Tjian R., Doudna J.A. (2016). Two Distinct Rnase Activities of Crispr-C2c2 Enable Guide-RNA Processing and RNA Detection. Nature.

[116] Shinoda H., Taguchi Y., Nakagawa R., Makino A., Okazaki S., Nakano M., Muramoto Y., Takahashi C., Takahashi I., Ando J. (2021). Amplification-Free RNA Detection with Crispr–Cas13. Commun. Biol..

[117] Wang Y., Xue T., Wang M., Ledesma-Amaro R., Lu Y., Hu X., Zhang T., Yang M., Li Y., Xiang J. (2022). Crispr-Cas13a Cascade-Based Viral RNA Assay for Detecting SARS-CoV-2 and Its Mutations in Clinical Samples. Sens. Actuators B Chem..

[118] Paige J.S., Nguyen-Duc T., Song W., Jaffrey S.R. (2012). Fluorescence Imaging of Cellular Metabolites with RNA. Science.

[119] Paige J.S., Wu K.Y., Jaffrey S.R. (2011). RNA Mimics of Green Fluorescent Protein. Science.

[120] Ying Z.-M., Wu Z., Tu B., Tan W., Jiang J.-H. (2017). Genetically Encoded Fluorescent RNA Sensor for Ratiometric Imaging of Microrna in Living Tumor Cells. J. Am. Chem. Soc..

[121] Fozouni P., Son S., de León Derby M.D., Knott G.J., Gray C.N., D’Ambrosio M.V., Zhao C., Switz N.A., Kumar G.R., Stephens S.I. (2021). Amplification-Free Detection of SARS-CoV-2 with Crispr-Cas13a and Mobile Phone Microscopy. Cell.

[122] Arizti-Sanz J., Freije C.A., Stanton A.C., Petros B.A., Boehm C.K., Siddiqui S., Shaw B.M., Adams G., Kosoko-Thoroddsen T.S.-F., Kemball M.E. (2020). Streamlined Inactivation, Amplification, and Cas13-Based Detection of SARS-CoV-2. Nat. Commun..

[123] Patchsung M., Jantarug K., Pattama A., Aphicho K., Suraritdechachai S., Meesawat P., Sappakhaw K., Leelahakorn N., Ruenkam T., Wongsatit T. (2020). Clinical Validation of a Cas13-Based Assay for the Detection of SARS-CoV-2 RNA. Nat. Biomed. Eng..

[124] Ke Y., Huang S., Ghalandari B., Li S., Warden A.R., Dang J., Kang L., Zhang Y., Wang Y., Sun Y. (2021). Hairpin-Spacer Crrna-Enhanced Crispr/Cas13a System Promotes the Specificity of Single Nucleotide Polymorphism (SNP) Identification. Adv. Sci..

[125] World Health Organization (2020). Laboratory Testing for Coronavirus Disease 2019 (COVID-19) in Suspected Human Cases: Interim Guidance, 2 March 2020.

[126] Feng W., Newbigging A.M., Le C., Pang B., Peng H., Cao Y., Wu J., Abbas G., Song J., Wang D.-B. (2020). Molecular Diagnosis of COVID-19: Challenges and Research Needs. Anal. Chem..

[127] Yan S., Ahmad K.Z., Warden A.R., Ke Y., Maboyi N., Zhi X., Ding X. (2021). One-Pot Pre-Coated Interface Proximity Extension Assay for Ultrasensitive Co-Detection of Anti-SARS-CoV-2 Antibodies and Viral RNA. Biosens. Bioelectron..

[128] Li S., Huang S., Ke Y., Chen H., Dang J., Huang C., Liu W., Cui D., Wang J., Zhi X. (2021). A Hipad Integrated with Rgo/Mwcnts Nano-Circuit Heater for Visual Point-of-Care Testing of SARS-CoV-2. Adv. Funct. Mater..

[129] Wei Y., Tao Z., Wan L., Zong C., Wu J., Tan X., Wang B., Guo Z., Zhang L., Yuan H. (2022). Aptamer-Based Cas14a1 Biosensor for Amplification-Free Live Pathogenic Detection. Biosens. Bioelectron..

[130] Ge H., Wang X., Xu J., Lin H., Zhou H., Hao T., Wu Y., Guo Z. (2021). A Crispr/Cas12a-Mediated Dual-Mode Electrochemical Biosensor for Polymerase Chain Reaction-Free Detection of Genetically Modified Soybean. Anal. Chem..

